# Interactions of *Escherichia coli* molecular chaperone HtpG with DnaA replication initiator DNA

**DOI:** 10.1007/s12192-015-0623-y

**Published:** 2015-08-06

**Authors:** Anna M. Grudniak, Katarzyna Markowska, Krystyna I. Wolska

**Affiliations:** Department of Bacterial Genetics, Institute of Microbiology, Faculty of Biology, University of Warsaw, Miecznikowa 1, 02-096 Warsaw, Poland

**Keywords:** Heat shock proteins, HtpG, DnaA, Interaction

## Abstract

The bacterial chaperone high-temperature protein G (HtpG), a member of the Hsp90 protein family, is involved in the protection of cells against a variety of environmental stresses. The ability of HtpG to form complexes with other bacterial proteins, especially those involved in fundamental functions, is indicative of its cellular role. An interaction between HtpG and DnaA, the main initiator of DNA replication, was studied both in vivo, using a bacterial two-hybrid system, and in vitro with a modified pull-down assay and by chemical cross-linking. In vivo, this interaction was demonstrated only when *htpG* was expressed from a high copy number plasmid. Both in vitro assays confirmed HtpG–DnaA interactions.

## Introduction

Hsp90 family proteins are highly conserved molecular chaperones that constitute up to 1–2 % of cellular proteins under normal physiological conditions (Chen et al. 2006). The cytoplasmic high-temperature protein G (HtpG) is a bacterial Hsp90 homolog found in most Eubacteria species (Buchner 2010; Chen et al. 2006; Gross 1996, Motojima-Miyazaki et al. 2010). Genest et al. identified a region in Hsp90 important for client binding in *E. coli* HtpG and suggested an evolutionary divergence in the mechanism of client interaction by bacterial and yeast Hsp90 (Genest et al. 2013) Furthermore, in contrast to the essential nature of eukaryotic Hsp90, deletion of *htpG* genes is not lethal to bacterial cells and only results in impaired growth at high temperatures (Buchner 2010; Grudniak et al. 2013). HtpG acts as a chaperone enabling the optimum folding of newly synthesized cellular proteins under stress conditions (Hartl 1996; Thomas and Baneyx 2000).

The HtpG-mediated folding mechanism does not involve co-chaperones and the identity of most of the substrate proteins remains unknown (Buchner 2010; Bukau and Horwich 1998; Zuehlke and Johnson 2010). The cyanobacterial HtpG has been shown to be involved in preventing protein aggregation, transporting polypeptides, and stabilizing a protein required for the assembly of phycobilisomes (Buchner 2010; Lindquist and Craig 1988). It has also been suggested that HtpG might be involved in regenerating the supply of ribosomal protein L2, essential for ribosome biogenesis, when L2 synthesis in the cell is reduced (Motojima-Miyazaki et al. 2010). In *Escherichia coli*, HtpG has been shown to interact with the DnaK/DnaJ/GrpE chaperone complex (Doyle et al. 2007).

The active form of HtpG is a dimer. The protein contains three highly conserved domains: an N-terminal domain (NTD), middle domain (MD), and C-terminal domain (CTD). The NTD contains a nucleotide binding pocket and exhibits ATPase activity. This domain is also capable of temporary dimerization, which is important in the HtpG–substrate binding cycle (Neckers et al. 2009). Genest et al. identified a region of HtpG that is important for ATP-dependent protein remodeling by HtpG in vitro and, more specifically, for client protein binding. The client binding region of HtpG was identified as comprised of residues in the middle and C-terminal domains that are on the surface of HtpG at the base of the cleft formed by the two protomers of a dimer. This region probably is responsible for interaction with multiple client proteins (Genest et al. 2011, Genest et al. 2013). In some HtpGs, the MD contains two subdomains which cooperate with the NTD in ATP hydrolysis (Buchner 2010; Huai et al. 2005; Neckers et al. 2009). Due to the presence of a four α-helix bundle motif, the CTD is responsible for dimerization of the protein (Harris et al. 2004). In solution, the native protein can adopt both open and closed conformations, with the former being predominant. Further conformational changes occur as a result of nucleotide (ATP or ADP) and substrate binding (Southworth and Agard 2008).

The DnaA protein is involved in the initiation of bacterial chromosomal DNA replication. It is a 52-kDa product of the *dnaA* gene (Schumann 2006) and is highly conserved in most Eubacteria species (Ozaki and Katayama 2009; Messer 2002). Like other initiator proteins, DnaA is capable of DNA binding and belongs to the AAA^+^ protein family (Leonard and Grimwade 2010). The activity of proteins in this family is regulated by ATP binding and hydrolysis. DnaA is also involved in the replication of several plasmids and acts as a transcription factor (Messer and Weigel, 1997; Ozaki and Katayama, 2009). This protein is characterized by a very high affinity for both ADP and ATP, but it is only active and capable of specifically binding DNA when bound to the latter (Bach and Skarstad 2008). Systems promoting the regeneration of DnaA-ATP complexes, necessary for subsequent rounds of replication, require interaction between DnaA-ADP and membrane phospholipids, as well as the involvement of two chromosomal DNA sites, known as DnaA-reactivating sequence (DARS), in the nucleotide exchange (Leonard and Grimwade 2010). The participation of the other chaperones proteins, DnaK and GrpE, in the activation of DnaA was previously shown (Hupp and Kaguni 1993; Hwang and Kaguni 1991).

In this study, we describe a previously unrecognized interaction between chaperone HtpG and replication initiator DnaA of *E. coli* that was demonstrated using both in vivo and in vitro experimental systems.

## Materials and methods

### Media, enzymes, and chemicals

Luria-Bertani (LB) or nutrient broth (for bacterial cultures and plating) and suspension medium (for dilution of cultures) were prepared as described by Miller (1972). Restriction enzymes, T4 DNA ligase, and DNA and protein markers were purchased from Fermentas and used as recommended by the manufacturer. Kits for genomic DNA purification and plasmid DNA preparation were supplied by A&A Biotechnology, Poland. Ni–nitrilotriacetic acid (NTA) agarose was purchased from Qiagen. The selective antibiotics ampicillin and kanamycin were used at 50 and 30 μg/ml, respectively. All chemicals and reagents were obtained from Sigma-Aldrich, unless stated otherwise. Oligonucleotides were synthesized by Genomed, Poland.

### Bacterial two-hybrid system

A bacterial two-hybrid system based on chimeric operator recognition, designed to study protein homo/heterodimerization in *E. coli* (Di Lallo et al. 2001), was used to examine HtpG–DnaA interactions in vivo. The following bacterial strains and plasmids were employed: *E. coli* R721 pcI_434_-*htpG*/pcI_P22_-*dnaA* and *E. coli* R721 pcI_434_-*dnaA*/pcI_P22_-*htpG*. Plasmids pcI_434_ and pcI_P22_ encode the N-terminal domains of the phage 434 and P22 repressors, respectively. The *dnaA*-coding sequence was amplified by PCR from *E. coli* K12 genomic DNA using primers dnaA1 (5′ GCGTCGACCGTGTCACTTTCGCTTTG 3′) and dnaA2 (5′ GCGGATCCTTACGATGACAATGTTC 3′). The *htpG*-coding sequence was amplified from the same DNA template using primers htpG3 (5′ GCGTCGACCATGAAAGGACAAGAAAC 3′) and htpG4 (5′ GCGGATCCTCAGGAAACCAGCAGCT 3′). All primers carried 5′ extensions including restriction endonuclease cleavage sites (underlined). To prepare the test constructs, the amplified DNA fragments were digested with restriction endonucleases and cloned into the pcI plasmids using the compatible *Sal*I and *Bam*HI sites. The recombinant plasmids pcI_434_-*htpG*, pcI_434_-*dnaA*, pcI_P22_-*dnaA*, and pcI_P22_-*htpG* were used to transform *E. coli* strain R721 (71/18*glpT*::O-P_434/P22_*lacZ*) which harbors the 434-P22 chimeric operator upstream of the *lacZ* gene (Di Lallo et al. 2001). *E. coli* R721 cells transformed with only one plasmid (pc22- or pc434-derived) were used as a negative control, and *E. coli* R721 containing plasmids pcI_434_434 and pcI_P22_434 was used as a positive control. The β-galactosidase activity produced by each strain was assayed in cultures grown to an A_600_ of 0.5 in LB medium supplemented with 0.1 mM isopropyl-β-d-thiogalactopyranoside (IPTG), as described by Miller (1972). Standard microbiological and recombinant DNA techniques were performed as described by Miller (1972) and Sambrook and Russell (2001), respectively.

### Cloning, expression, and purification of recombinant HtpG and DnaA

*E. coli* Top10 [F^¯^*mcrA* Δ(*mrr*-*hsdRMS*-*mcrBC*) φd/*lacZ* ΔM15 Δ*lacX74 deoR recA1 araD139* Δ(*araA*-*leu*) *7697 galU galK rpsL endA1 nupG*] (Stratagene) were used in cloning experiments and *E. coli* BL21 (DE3) [F^¯^*ompT gal dcm lon hsdS*_B_ (r_B_^−^ m_B_^−^) λ(DE3)] (Novagen) were used in expression experiments. These strains and their derivatives were grown in LB broth medium supplemented with kanamycin (25 or 30 μg/ml). The genes *htpG* (nt 2729622 to 2732195 in the *E. coli* K12 genome) and *dnaA* (nt 3880349 to 3881752 in the *E. coli* K12 genome) were amplified by PCR from *E. coli* K12 chromosomal DNA. The *dnaA* coding sequence was amplified using primers dnaA3 (5′ ATCGCATATGGTGTCACTTTCGCTTTGGCA 3′) and dnaA4 (5′ CCATGGATCCGCACGATGACAATGTTCTGA 3′). The *htpG* coding sequence was amplified using primers htpG1 (5′ ATCGCATATGAAAGGACAAGAAACTCGTGG 3′) and htpG2 (5′ CCATGGATCCTCAGGAAACCAGCAGCTGGT 3′). All primers carried 5′ extensions including restriction endonuclease cleavage sites (underlined). The PCRs were performed using HotStar HiFidelity polymerase (Qiagen) according to the manufacturer’s recommendations. Following cleavage with *Bam*HI and *Nde*I, the DNA fragment encoding *dnaA* was cloned into the corresponding sites of vector pET28a (Novagen) to produce the construct pAG-A1m, while the DNA fragment encoding *htpG* cleaved with the same enzymes was cloned into the corresponding sites of pET28a (Novagen) to produce the construct pAG-B2. To express and purify His-tagged forms of HtpG and DnaA, single fresh transformant colonies of *E. coli* BL21 (DE3)(pAG-G1) and *E. coli* BL21 (DE3)(pAG-A2), respectively, were used to inoculate 100 ml lots of LB broth containing kanamycin. The cultures were incubated at 37 °C with shaking to an A_600_ of 0.6 and then IPTG was added to a final concentration of 1 mM. Incubation was continued at 30 °C for an additional 12 h. The cells were then collected by centrifugation and the bacterial pellet resuspended in 10 ml of buffer (50 mM NaH_2_PO_4_, 300 mM NaCl, 1 mM imidazole, 10 mM 2-mercaptoethanol, 0.1 % Tween 20, 55 μM phenylmethylsulfonyl fluoride -PMSF), which was supplemented with one tablet of EDTA-free complete Mini protease inhibitor cocktail (Roche Diagnostics) in the case of HtpG. The cells were disrupted by sonication (MSE 150 disintegrator, frequency 20 kHz, amplitude 12–16 μm) and the cell debris pelleted by centrifugation at 40,000×*g* for 1 h at 4 °C. The supernatants were applied to 3-ml Ni–nitrilotriacetic acid (NTA) agarose columns that had been equilibrated with 100 ml of cell suspension buffer. Once all liquid had passed through the columns, they were then washed with 250 ml of wash buffer (50 mM NaH_2_PO_4,_ 300 mM NaCl, 20 mM imidazole, 10 % glycerol) and the proteins were eluted by a gradient of imidazole (0.1–1 M) in wash buffer. HtpG was eluted at 0.25 to 0.3 M imidazole and DnaA at 0.75 to 1 M imidazole. The homogeneity of the protein preparations was verified by electrophoresis on a 12.5 % SDS-PAGE gel followed by Coomassie blue staining (Katayama et al., 1988). The purified proteins were quantified using Bradford Reagent (Sigma-Aldrich) with bovine serum albumin (BSA) as the standard. The concentrations of the preparations were ~1.6 mg/ml for HtpG and ~1.2 mg/ml for DnaA.

### Modified pull-down assay

His-tagged DnaA (5 μM) in 50 μl of buffer A (50 mM Tris–HCl, pH 8.0, 0.3 M NaCl, 20 mM imidazole) was incubated with 5 μl of Ni–NTA agarose (Qiagen) for 3 h at 4 °C. The agarose beads were washed twice with buffer A and full length of HtpG (1.8 μM) was then added in 20 μl of buffer B (50 mM Tris–HCl, pH 7.8, 50 mM KCl, 5 mM 2-mercaptoethanol, 0.1 mM EDTA, 10 mM MgCl_2_, 10 % glycerol). After 30-min incubation at room temperature, the agarose beads were washed twice with 25 μl of buffer A and bound proteins were eluted with buffer A containing 250 mM imidazole for HtpG and 750 mM imidazole for DnaA in three 25-μl steps for each protein. In separate tests, nucleotides (2 mM ATP or ADP) were included in the incubation, wash, and elution buffers. All eluate and wash fractions were analyzed by SDS-PAGE, and the separated proteins were electroblotted onto polyvinylidene difluoride (PVDF) membrane (Millipore) and detected by reaction with rabbit polyclonal anti-HtpG antibody (Eurogentec, Belgium) at a dilution of 1:10,000, followed by anti-rabbit IgG (whole molecule) alkaline phosphatase-conjugated secondary antibody at a dilution of 1:15,000. Reactive protein bands on the blot were visualized using BCIP/NBT chromogenic substrate. In a set of reciprocal tests, His-tagged HtpG (5 μM) was immobilized on Ni–NTA agarose and incubated with full length of DnaA (1.8 μM) as described above. DnaA in the eluate and wash fractions was detected by immunoblotting with rabbit polyclonal anti-DnaA antibody (Eurogentec, Belgium) at a dilution of 1:1000. The reactivity of the primary antibodies with truncated variants of DnaA and the ATPase domain of HtpG was not significantly different from that observed with the full-length proteins. In another set of tests, His-tagged HtpG (5 μM) was immobilized on Ni–NTA agarose and incubated with DnaA variants (1.8 μM), and this method is previously described by Kędzierska et al. 2005.

### Chemical cross-linking

Purified His-tagged HtpG and DnaA were diluted in phosphate-buffered saline (PBS). Aliquots of 3 μl of each protein were then incubated with 0.5 μl of 0.1 % glutaraldehyde in a 50 μl bicine-buffered solution. After 30 min at 22 °C, cross-linking was stopped by adding 20 μl of ethanolamine and incubating for a further 20 min. The reactions were then mixed with SDS-PAGE loading buffer containing 0.5 % 2-mercaptoethanol and analyzed by electrophoresis on 12.5 % SDS-PAGE gels. The separated proteins were electroblotted onto PVDF membrane and detected by reaction with rabbit polyclonal anti-HtpG or anti-DnaA antibodies (Eurogentec, Belgium), followed by anti-rabbit IgG (whole molecule) alkaline phosphatase-conjugated secondary antibody. Reactive protein bands on the blot were visualized using BCIP/NBT chromogenic substrate.

## Results

Potential interactions between the proteins HtpG and DnaA were examined in vivo in *E. coli* cells, using a bacterial two-hybrid system including positive and negative controls, as described by Di Lallo et al. (2001). This system is based on a chimeric operator present on the chromosome of the *E. coli* strain R721. The genes encoding the proteins of interest are cloned in the two plasmids pcI_434_ and pcI_P22_ to produce translational fusions with the N-terminal domains of the phage 434 and P22 repressors, respectively. The recombinant plasmids are then introduced into *E. coli* R721, which has the 434-P22 chimeric operator upstream of the *lacZ* gene. If the two proteins under investigation interact, a functional repressor is formed and the expression of β-galactosidase is reduced. A positive interaction between proteins is scored when the β-galactosidase activity in the test strain is less than half that of strain R721 (Di Lallo et al. 2001). The results presented in Table 1 demonstrate that HtpG is capable of interacting with DnaA when the gene encoding the former is located on the high copy number plasmid pcI_P22_ and that encoding the latter is located on the low copy number plasmid pcI_434_. The ratio of the β-galactosidase activity of this strain to that of the untransformed strain R721 is 0.19, which indicates a strong interaction between these proteins. In physiological conditions in *E. coli* R721 strain, HtpG, and DnaA proteins are endogenous and β-galactosidase activity in Miller units was 5518.6 ± 205.8. The ratio of β-galactosidase activity in tested strain to R721 strain was 1 (Table 1), but in restrictive conditions when the cells were treated by temperature 42 °C for 2 h, β-galactosidase activity was decreased and in Miller units it was 3135.1 ± 69.9. The ratio was 0.57.

**Table 1:** Interaction between HtpG and DnaA estimated by measuring β-galactosidase activity in bacterial two-hybrid system

Strain	β-Galactosidase activity ± SD	Ratio β-galactosidase activity tested strain/R721
C+ (positive control)	1172.5 ± 92.1	0.21
C− (negative control)	4827.9 ± 674.7	0.87
*E. coli* R721	5518.6 ± 205.8	1
pcI_434_-*htpG*/pcI_P22_-*dnaA*	7752.6 ± 895.7	1.4
pcI_434_-*dnaA*/pcI_P22_-*htpG*	1072.6 ± 149.9	0.19

β-Galactosidase activity was calculated in Miller units. Each value represents the mean of a minimum of three separate determinations

**Fig. 1:**
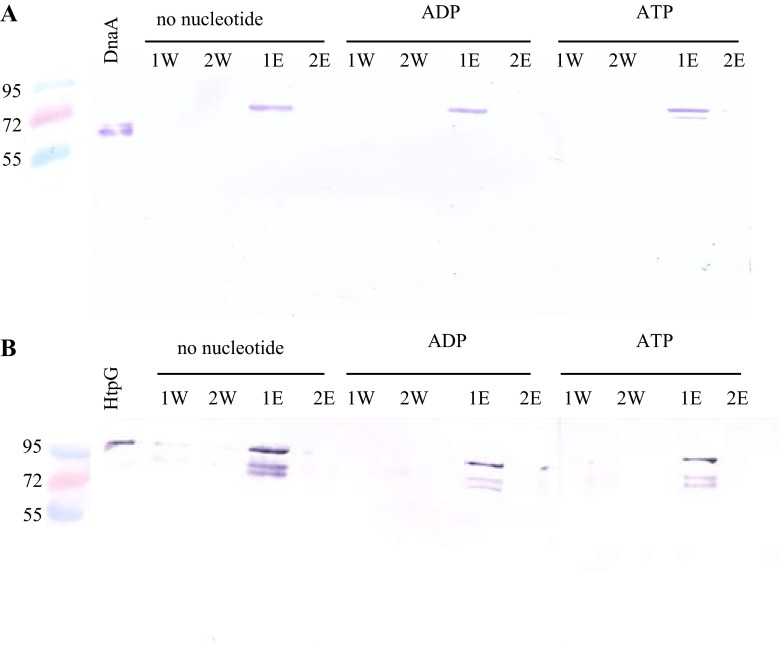
The in vitro interaction between HtpG and DnaA demonstrated in a modified pull-down assay. **a** His-tagged HtpG was immobilized on nickel–agarose beads and incubated with the full-length DnaA in the absence of nucleotides or with 2 mM ADP or ATP. The following samples were analyzed by SDS-PAGE and immunoblotting using an anti-DnaA antibody: control purified DnaA, unbound DnaA in two consecutive wash fractions (1W, 2W), and bound DnaA eluted by imidazole in two consecutive elution fraction (1E, 2E). **b** His-tagged DnaA was immobilized on nickel–agarose beads and incubated with the full-length HtpG in the absence of nucleotides ADP or ATP. The following samples were analyzed by SDS-PAGE and immunoblotting using an anti-HtpG antibody: control purified HtpG, unbound HtpG in two consecutive wash fractions (1W, 2W), and bound HtpG eluted with imidazole in two consecutive elution fractions (1E, 2E)

**Fig. 2:**
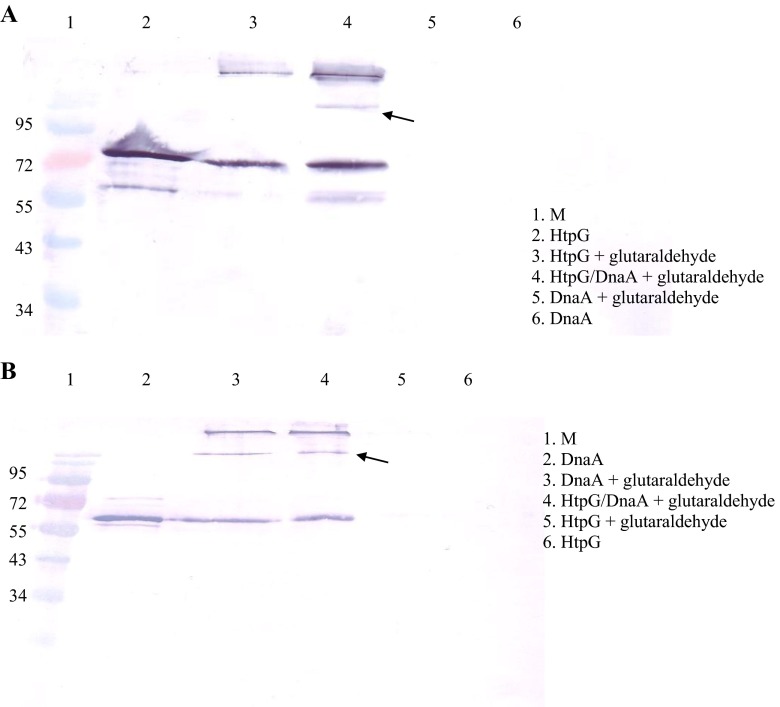
Interaction between HtpG and DnaA demonstrated in glutaraldehyde cross-linking assay. Representative Western blot with **a** antibodies against HtpG and **b** antibody against DnaA are presented. HtpG–DnaA complexes are indicated by arrows. M - protein size markers

To further investigate the interaction of HtpG and DnaA, His-tagged versions of these proteins were expressed and purified using Ni–NTA agarose affinity columns. Imidazole concentrations of 750 and 250 mM were required to elute these forms of DnaA and HtpG, respectively. In vitro interactions between recombinant HtpG and DnaA were examined using a previously described modified pull-down assay (Kędzierska et al. 2005). One of the His-tagged proteins (HtpG or DnaA) was immobilized on Ni–NTA agarose beads using appropriate conditions to ensure that the bed volume was fully saturated. Then the second protein (DnaA or HtpG, respectively) was added to the beads. Three different binding reactions were performed in both of the assay variants: (1) with no additional factors that might affect the interaction, (2) with added ADP, and (3) with added ATP. After a period of incubation, the proteins initially bound to the beads were eluted from the column using a buffer containing the required imidazole concentration. All eluent fractions were then separated by electrophoresis (SDS-PAGE) and Western blotted with the appropriate anti-DnaA or anti-HtpG antibody. The results demonstrated that HtpG interacts with DnaA in both variants of the assay. In the eluent fraction (1E) from the columns with immobilized HtpG (Fig. 1a) or DnaA (Fig. 1b), DnaA and HtpG were found, respectively. No significant differences in binding were observed with the addition of ADP or ATP to the reactions. The in vitro interaction between HtpG and DnaA was also examined by chemical cross-linking. Following treatment of HtpG and DnaA mixtures with glutaraldehyde, the reactions were analyzed by SDS-PAGE followed by Western blotting. An additional band observed on the membrane probed with anti-HtpG antibody represents a complex formed by HtpG and DnaA in the presence of the cross-linking agent (arrow in Fig. 2), which is further evidence of the interaction between these proteins. On the membrane with anti-DnaA antibody, complexes of the two proteins were also visible (Fig. 2b). However, the DnaA protein alone forms dimmers, trimers, or oligomers in the presence of a cross-linking agent which can create the difficulty to characterize the observed band in the identification of band representing cross-linked proteins (Felczak et al. 2005).

## Discussion

The DnaA protein is a key factor in the process of initiating bacterial chromosome replication. Its structure allows it to interact with DNA, with other DnaA monomers, and with other proteins. The chaperone HtpG, a member of the Hsp90 heat shock protein family, is vital for the normal functioning of prokaryotic cells under stress conditions, especially at high temperatures. Therefore, the interaction between HtpG and DnaA identified in this study is likely to be important for the survival of bacterial cells in the natural environment.

The association of HtpG and DnaA in vivo was demonstrated using a bacterial two-hybrid system. This interaction was only observed when the *htpG* gene was located on a high copy number plasmid, and the *dnaA* gene was located on a low copy number plasmid. It may be concluded that overproduction of the HtpG protein is required for this interaction to occur. Under normal physiological conditions, bacterial cells produce significantly larger amounts of DnaA than HtpG. The expression of *htpG* increases during heat shock, so it is likely that the interaction with DnaA occurs under stress conditions, when the cellular level of HtpG is raised. In this experimental system, interaction between these proteins caused a significant reduction in β-galactosidase activity in comparison with strain R721 containing no plasmids (activity ratio 0.19, where interactions are considered positive when the ratio is <0.5).

In vitro analyses using purified recombinant DnaA and HtpG confirmed the interaction between these proteins. The purification of bacterial DnaA is known to be problematic because of its tendency to aggregate and precipitate in the affinity column, which makes efficient elution difficult (Zawilak-Pawlik et al. 2006). Therefore, the use of denaturing conditions for purification is often suggested. However, this results in the destruction of the protein’s native structure necessary for in vitro functional analyses. In the present study, an immobilized metal ion affinity chromatography (IMAC) technique optimized for the purification of recombinant proteins under non-denaturing conditions was applied. This permitted the isolation of native, biologically active DnaA and HtpG polypeptides for the study of their interactions.

Binding to protein immobilized on Ni–NTA agarose beads verified the existence of interactions between HtpG and DnaA. The modified pull-down assay used here is highly sensitive and specific (Kędzierska et al. 2005). It requires the use of recombinant His-tagged proteins and carefully controlled conditions to prevent protein that is added to the reaction, and not directly immobilized, from binding to the beads. These conditions include both saturation of the bead with the appropriate amount of protein and reaction buffers that create the necessary environment for protein-protein interactions. Although three different binding reaction variants were tested, HtpG–DnaA interactions were observed irrespective of the presence of either ADP or ATP.

Despite the failure to observe any significant differences in the ability of DnaA and HtpG to form complexes in the presence or absence of nucleotides (ADP or ATP), both proteins are capable of binding and hydrolyzing ATP, and these activities strongly influence their normal functioning. No significant differences were observed, in the ability of DnaA and HtpG to form the abovementioned complexes in the presence or absence of either nucleotide. HtpG undergoes structural changes after binding ATP, and structural rearrangements also occur in the presence of ADP and after binding a substrate molecule. The closed conformation of HtpG that occurs after it binds ATP does not permit substrate protein binding. Therefore, the influence of ATP binding and hydrolysis on HtpG interactions with protein substrates remains unknown (Buchner 2010). DnaA forms complexes with both ADP and ATP. The binding and hydrolysis of ATP is crucial during this protein’s involvement in replication initiation, i.e., in its association with DNA and in the separation of the strands of the DNA double helix in the *oriC* region of the bacterial chromosome. However, our findings indicate that the binding of nucleotides by DnaA is not required for the formation of complexes with HtpG. The identified interactions between DnaA and HtpG could be an example of typical chaperone-to-substrate binding. In such a case, the NTD or MD of the HtpG protein is likely to be involved in the interaction (Buchner 2010). Even though HtpG exhibits ATPase activity, ATP is not required for its chaperone function under physiological conditions. The NTD of this protein is responsible for the ATPase activity, but under non-stress conditions, it is hidden by part of the MD (Nemoto et al., 2001). So far, the exact substrate binding site and the substrate binding cycle of HtpG remain uncharacterized. More is known about this process in eukaryotic Hsp90s, but it is not valid to extrapolate these findings to the analogous chaperones in prokaryotic cells. Therefore, a full description of the pathway of DnaA–HtpG complex formation awaits further experimental evidence. Summarizing, it was shown that HtpG–DnaA interaction is not ATP-dependent.

Interactions between HtpG and DnaA were also studied by chemical cross-linking. The use of glutaraldehyde, a homobifunctional cross-linker, stabilized complexes of these two proteins formed in vitro. Glutaraldehyde forms permanent covalent bonds with the amine groups of basic amino acids (most commonly lysine) in protein molecules. For permanent bonds to be formed, it has been estimated that the minimum lysine content of the interacting proteins needs to be approx. 7 %. The lysine contents of HtpG and DnaA are 7.4 and 4.92 %, respectively, but despite this, Western blot analysis using a specific anti-HtpG antibody confirmed the presence of heterologous complexes formed by HtpG and DnaA. On an identical blot probed with anti-DnaA antibody, an additional streak was also observed, probably representing a DnaA–HtpG complex. However, self-oligomerization of DnaA via its N-terminal domain (Felczak et al. 2005; Simmons et al. 2003) can produce crosslinked complexes, which complicates the interpretation of results obtained using the anti-DnaA antibody. In a sample containing only the DnaA protein and the cross-linker, we observed self-oligomerization of DnaA, which makes it more difficult to interpret the results gathered with anti-DnaA serum. The N-terminal domain of DnaA is involved in this oligomerization (Felczak et al. 2005; Simmons et al. 2003).

In conclusion, the interaction between HtpG and DnaA identified in this study suggests the involvement of HtpG in DnaA stabilization, although this chaperone may also participate in the main physiological function of DnaA, namely the initiation of chromosomal DNA replication.
